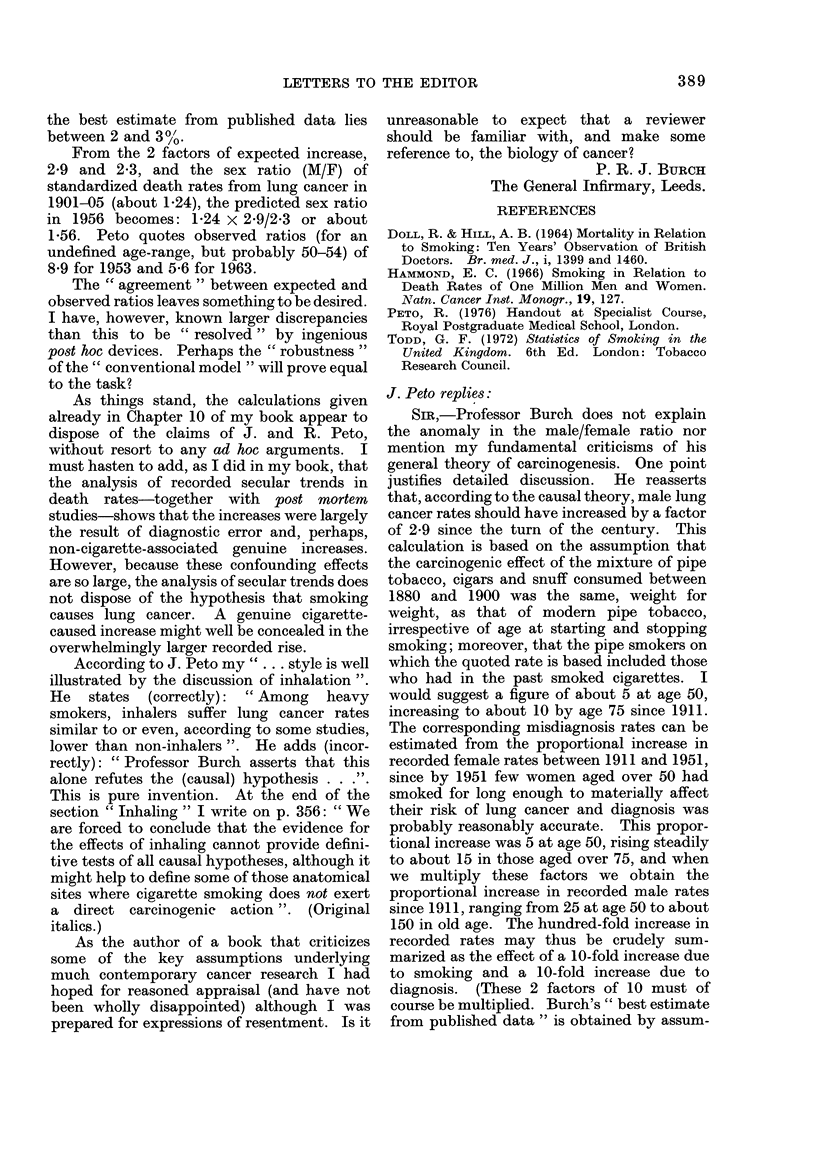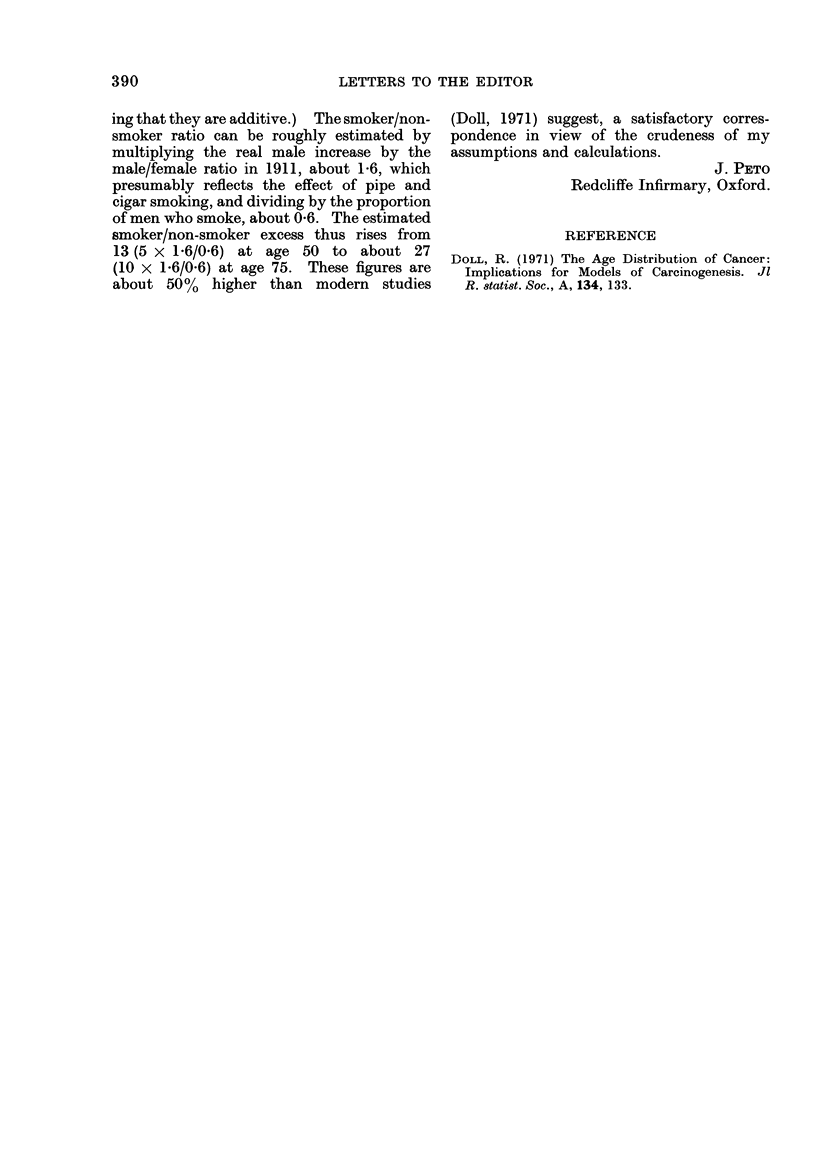# J. Peto replies

**Published:** 1977-03

**Authors:** J. Peto


*J. Peto replies:*

Sir,—Professor Burch does not explain the anomaly in the male/female ratio nor mention my fundamental criticisms of his general theory of carcinogenesis. One point justifies detailed discussion. He reasserts that, according to the causal theory, male lung cancer rates should have increased by a factor of 2·9 since the turn of the century. This calculation is based on the assumption that the carcinogenic effect of the mixture of pipe tobacco, cigars and snuff consumed between 1880 and 1900 was the same, weight for weight, as that of modern pipe tobacco, irrespective of age at starting and stopping smoking; moreover, that the pipe smokers on which the quoted rate is based included those who had in the past smoked cigarettes. I would suggest a figure of about 5 at age 50, increasing to about 10 by age 75 since 1911. The corresponding misdiagnosis rates can be estimated from the proportional increase in recorded female rates between 1911 and 1951, since by 1951 few women aged over 50 had smoked for long enough to materially affect their risk of lung cancer and diagnosis was probably reasonably accurate. This proportional increase was 5 at age 50, rising steadily to about 15 in those aged over 75, and when we multiply these factors we obtain the proportional increase in recorded male rates since 1911, ranging from 25 at age 50 to about 150 in old age. The hundred-fold increase in recorded rates may thus be crudely summarized as the effect of a 10-fold increase due to smoking and a 10-fold increase due to diagnosis. (These 2 factors of 10 must of course be multiplied. Burch's " best estimate from published data " is obtained by assuming that they are additive.) The smoker/non-smoker ratio can be roughly estimated by multiplying the real male increase by the male/female ratio in 1911, about 1·6, which presumably reflects the effect of pipe and cigar smoking, and dividing by the proportion of men who smoke, about 0·6. The estimated smoker/non-smoker excess thus rises from 13 ($5 \times 1{\cdot}6/0{\cdot}6$) at age 50 to about 27 ($10 \times 1{\cdot}6/0{\cdot}6$) at age 75. These figures are about 50% higher than modern studies (Doll, 1971) suggest, a satisfactory correspondence in view of the crudeness of my assumptions and calculations.

J. Peto
Redcliffe Infirmary, Oxford.

### REFERENCE

Doll, R. (1971) The Age Distribution of Cancer: Implications for Models of Carcinogenesis. *Jl R. statist. Soc.*, A, **134**, 133.